# Supplementary material for: Cardiac findings in infants with in utero exposure to Zika virus – a follow up longitudinal study
Source: PLoS Negl Trop Dis. 2026 Feb 12;20(2):e0014009. doi: 10.1371/journal.pntd.0014009 (PMC12919927; doi:10.1371/journal.pntd.0014009)
Supplement: Attachment — Submitted filename: Zika revision comments.docx [file pntd.0014009.s002.docx]

Please submit your revised manuscript within 60 days Dec 09 2025 11:59PM.

Please include the following items when submitting your revised manuscript:

* A rebuttal letter that responds to each point raised by the editor and reviewer(s). You should upload this letter as a separate file labeled 'Response to Reviewers'. This file does not need to include responses to any formatting updates and technical items listed in the 'Journal Requirements' section below.
* A marked-up copy of your manuscript that highlights changes made to the original version. You should upload this as a separate file labeled 'Revised Manuscript with Track Changes'.
* An unmarked version of your revised paper without tracked changes. You should upload this as a separate file labeled 'Manuscript'.


**Additional Editor Comments:**

I advocate the authors to answer specifically all of the comments since the opinion of these reviewers will be seeked for final decision


**Journal Requirements:**

1) Please provide an Author Summary. This should appear in your manuscript between the Abstract (if applicable) and the Introduction, and should be 150-200 words long. The aim should be to make your findings accessible to a wide audience that includes both scientists and non-scientists. Sample summaries can be found on our website under Submission Guidelines:
<https://journals.plos.org/plosntds/s/submission-guidelines#loc-parts-of-a-submission>

2) Please upload all main figures as separate Figure files in .tif or .eps format. For more information about how to convert and format your figure files please see our guidelines: 
<https://journals.plos.org/plosntds/s/figures>

3) Tables should not be uploaded as individual files. Please remove these files and include the Tables in your manuscript file as editable, cell-based objects. For more information about how to format tables, see our guidelines:
<https://journals.plos.org/plosntds/s/tables>

4) In the online submission form, you indicated that "The data that support the findings of this study are available from the corresponding author upon reasonable request.". All PLOS journals now require all data underlying the findings described in their manuscript to be freely available to other researchers, either
1. In a public repository
2. Within the manuscript itself
3. Uploaded as supplementary information.
This policy applies to all data except where public deposition would breach compliance with the protocol approved by your research ethics board. If your data cannot be made publicly available for ethical or legal reasons (e.g., public availability would compromise patient privacy), please explain your reasons by return email and your exemption request will be escalated to the editor for approval. Your exemption request will be handled independently and will not hold up the peer review process, but will need to be resolved should your manuscript be accepted for publication. One of the Editorial team will then be in touch if there are any issues.

**Methods**


-Are the objectives of the study clearly articulated with a clear testable hypothesis stated?

-Is the study design appropriate to address the stated objectives?

-Is the population clearly described and appropriate for the hypothesis being tested?

-Is the sample size sufficient to ensure adequate power to address the hypothesis being tested?

-Were correct statistical analysis used to support conclusions?

-Are there concerns about ethical or regulatory requirements being met?

Reviewer #1: Acceptable

Reviewer #2: The objective of the study is clearly articulated in the final paragraph of the introduction, and the study design is generally appropriate for addressing this aim. However, additional methodological details would strengthen the manuscript and improve clarity.

• The description of the study sample could be expanded. Specifically, in lines 129–132, it should be clarified whether any positive result of the referenced test was sufficient to determine maternal ZIKV exposure.

The following citation was added to support the statement that any positive result of the referenced test was sufficient to determine maternal ZIKV exposure.

Brasil P, Vasconcelos Z, Kerin T, Gabaglia CR, Ribeiro IP, Bonaldo MC, et al. Zika virus vertical transmission in children with confirmed antenatal exposure. Nat Commun. 2020 July 14;11(1):3510.

• At line 137, the authors should explain why some children did not return for the follow-up echocardiogram.

The following statement was added for clarity: The primary reasons for loss to follow-up were attributed to socioeconomic barriers commonly faced by low-income and socially vulnerable populations.

• Lines 137–140: Please specify how many children were excluded due to other congenital infections and genetic syndromes.

The following statement was added for clarity: Overall, no children were excluded since patients were referred to IFF-FIOCRUZ after meeting all inclusion criteria: primary ZIKV exposure without an underlying genetic syndrome, and indication for cardiovascular assessment.

• While ethical approval is mentioned, the approval reference numbers from each IRB (INI, IFF/FIOCRUZ, and UCLA) should be provided.

The approval reference numbers were added: Brazilian National Institute of Infectious Diseases (INI and IFF/FIOCRUZ #2675616.0.0000.5269) and UCLA (# 17-000104).

• Were the pediatric cardiologists blinded to ZIKV exposure status?

The following statement was added for clarity: Blinding of cardiologists was not possible, particularly in children with microcephaly, where clinical features were evident. Further, echocardiographic evaluations were conducted during a time of increased clinical awareness of ZIKV-related findings.

• Were all prenatal check-ups conducted at the Pediatric Infectious Disease Clinic, or were some patients referred from other centers?

The following statement was added for clarity: Although prenatal care and deliveries frequently occurred at outside facilities, families were referred to IFF-FIOCRUZ for postnatal follow-up related to ZIKV exposure and comprehensive evaluation, including echocardiography.

• Were deliveries attended exclusively at the study hospital, or were children born in other hospitals also eligible for referral and inclusion?

The following statement was added for clarity: Although prenatal care and deliveries frequently occurred at outside facilities, families were referred to IFF-FIOCRUZ for postnatal follow-up related to ZIKV exposure and comprehensive evaluation, including echocardiography.

Reviewer #3:

While the research topic is interesting and relevant, the research question is not clearly defined. It remains ambiguous whether the main goal is to document the frequency of cardiac abnormalities among microcephalic infants, to compare microcephalic vs normocephalic infants, or to investigate or establish a relationship between antenatal ZIKV infection and congenital heart defects (CHD). The authors should clarify the primary and secondary objectives explicitly. The absence of a non-ZIKV-exposed control group seriously limits the capacity to establish whether the prevalence of cardiac findings is truly higher than in the general population. Selection bias is possible since only 169/216 (78%) underwent echocardiography, and no comparison is provided between included and excluded infants. Analytical methods are broadly acceptable (chi-squared, logistic regression), but additional analyses would strengthen the results: (i) provide univariate and multivariate prevalence estimates with crude percentages; (ii) include interaction terms (e.g., microcephaly × trimester of infection); and (iii) present sensitivity analyses stratified by age at echocardiography, as PDA and other lesions may be physiological in early life.

We clarified the research question and objectives in the Introduction section:

“In this longitudinal assessment, our research questions were: what is the prevalence and spectrum of structural cardiac abnormalities; do these risks differ by cephalic status at birth; and what proportion of anomalies persist on repeat echocardiography? Accordingly, our objectives were to (i) estimate the prevalence of any structural cardiac abnormalities in early infancy; (ii) compare prevalence and abnormality patterns between MC and normocephalic (NC) infants; (iii) characterize lesion-specific frequencies; (iv) determine the proportion of defects that resolve versus persist on follow-up; and (v) explore clinical and perinatal predictors of cardiac abnormalities.”

We also added a thorough discussion about the limitations delineated above, as well as the ways we worked to address them in the Discussion:

“However, there are several limitations to acknowledge. First, we lacked a non-exposed control group, as this was a clinic-based cohort assembled during a public health emergency. Notably, recruiting controls and screening asymptomatic women was not technically feasible during the epidemic given that healthcare resources were heavily strained by the large number of symptomatic referrals. Therefore, our estimates of about 18% abnormal echocardiograms overall, and a higher abnormal rate among MC than NC infants, are benchmarked to population norms rather than infants imaged under identical protocols. Second, selection/referral bias is possible because of 216 exposed infants with postnatal follow-up, 169 (78%) underwent echocardiography, and recruitment at a tertiary pediatric center may result in more complex phenotypes. Third, we acknowledge that follow-up was incomplete, which might have resulted in an underpowered analysis of persistent CV abnormalities: of the 30 infants with initial abnormalities, 24 returned and roughly one quarter had persistent lesions. Additionally, distinguishing physiologic from pathologic findings is challenging given age heterogeneity at imaging (initial 86 ± 80 days; repeat 188 ± 169 days). Finally, we recognize that none of the defects were found to have major clinical repercussions in early childhood, thus the impact of this increased rate of cardiac lesions still needs to be explored.

We designed our follow up study with these limitations in mind. Despite the absence of a non-exposed control group, we included an internal MC-NC comparison under identical protocols, complemented by multivariable analyses adjusting for gestation, sex, birth weight, delivery mode, trimester of infection, maternal age, hypertension, and diabetes, which still supported an association between MC and cardiac anomalies. Echocardiography was proactively offered to all patients with laboratory-confirmed ZIKV exposures, thus reducing selective imaging by symptom severity. Moreover, imaging acquisition and interpretation were performed by pediatric cardiologists using standardized protocols. Lastly, to minimize the challenges of differentiating between physiologic and pathologic findings, we standardized and prespecified physiologic windows (e.g., PDA in term infants ≤ 15 days and in preterm infants ≤ 3 months; PBS normal in preterm) and performed sensitivity analyses incorporating age at echocardiography. Although these measures do not completely eliminate the limitations described above, they improve the study’s internal validity and highlight the increased prevalence of structural cardiac abnormalities in ZIKV-exposed infants, especially among those with MC.”

Statistics:

i) We calculated multivariate prevalence using the GENLIN command in SPSS. The revised results list the multivariate prevalence (NC: 12%, MC: 31%).
ii) We performed the logistic regression with an interaction between trimester of infection and microcephaly. The interaction term was not significant (p>0.05).
iii) As age at echocardiography was a continuous variable, performing stratification required dividing age into groups. To this end, we calculated the median age, which was 3 months and 13 days. To stratify by age, we categorized each participant as above or below the median. As shorthand, we will refer to those above the median age as “late” group and those below the median age as the “early” group. When the groups are pooled, microcephaly is associated with higher risk of abnormalities. However, as the reviewer pointed out, this finding raises the question of whether the association was driven by PDA and other lesions that are physiological early in life. To investigate this, we performed logistic regression restricting the analysis to the late group. The results indicated that among those with echocardiography performed later, microcephaly was associated with higher risk of abnormalities: OR: 6.0 (1.03-34.94), p=0.046. We believe this result provides evidence supporting the robustness of the association by age. The revised manuscript describes this sensitivity analysis.

**Results**


-Does the analysis presented match the analysis plan?

-Are the results clearly and completely presented?

-Are the figures (Tables, Images) of sufficient quality for clarity?

Reviewer #1: Need analysis based on time of follow-up, which is equivalent to infant age.

The mean (sd) age in days at follow-up was 180 (174) days for NC infants and 196 (168) for MC. To investigate whether the interval was significantly different between MC and NC infants, we compared the age of follow-up between the groups using a t-test. The results indicated that the interval was similar in MC and NC infants (p=0.88). We believe that this increases confidence that there is not a significant bias in the age at follow-up.

Reviewer #2: 
• Clarification is needed regarding the number of symptomatic patients and whether any required medical or surgical treatment.
• Table 3 should include crude ORs so that readers can assess how the estimates changed after adjustment in the multivariate logistic regression.

The following statement was added for clarity: There were no severe defects (those requiring immediate medical or surgical intervention)

Crude ORs were included in Table 3.

Reviewer #3:

Table 1 should include statistical tests (p-values) to facilitate comparison between microcephalic and normocephalic infants.

Table 1 was revised to include p values

Table 2 appears structured to explore risk factors for microcephaly, but the more intuitive approach would be to present cardiac abnormalities as the outcome in the columns, with associated factors (microcephaly, SGA, prematurity, trimester of infection, sex) in rows. The table should also indicate denominators and the number of patients affected by each anomaly, not only the number of anomalies. For example: 'VSD: 6 patients (20% of the 30 abnormal patients)'.

Table 2: We reviewed and attempted the suggested formatting. However, we believe it makes the data more difficult to interpret, and we respectfully request to retain the original format. To support clarity, we have added denominators within the table, as requested, to facilitate interpretation.

Figure 1 shows the flow of patients but does not allow direct evaluation of potential selection bias. The persistence of anomalies is based on extremely small numbers (e.g., 4 MC vs 2 NC children), making percentage comparisons misleading. The results should be described with greater caution.

Figure 1 was revised to reflect the total number of patients, rather than the percentage.

**Conclusions**


-Are the conclusions supported by the data presented?

-Are the limitations of analysis clearly described?

-Do the authors discuss how these data can be helpful to advance our understanding of the topic under study?

-Is public health relevance addressed?

Reviewer #1: Need analysis based on time of follow-up, which is equivalent to infant age.

The mean (sd) age in days at follow-up was 180 (174) days for NC infants and 196 (168) for MC. To investigate whether the interval was significantly different between MC and NC infants, we compared the age of follow-up between the groups using a t-test. The results indicated that the interval was similar in MC and NC infants (p=0.88). We believe that this increases confidence that there is not a significant bias in the age at follow-up.

Reviewer #2:

• The conclusions tend to overstate the results. Clinical significance is not clearly discussed; it is only briefly mentioned that the anomalies did not have major repercussions. This point should be elaborated and integrated into the Results and Discussion sections.

The following statement was added for clarity: Importantly, none of the cardiac lesions were hemodynamically significant, and no child required medical or invasive intervention from a cardiac perspective. Therefore, the clinical implications of these echocardiographic findings remain uncertain, as it is not yet clear whether they translate into meaningful differences in patient outcomes.

• At lines 273–275, the statement “over 75% of children” is misleading. The data suggest that 3.6% of the original cohort, or 20% of children requiring a follow-up echocardiogram, did not return. The sentence should be revised accordingly.

We understand how that can be misleading. We have addressed this in the Discussion: On repeat assessment, among infants with abnormal initial echocardiograms who returned (24/30), 18 (75.0%) normalized; at the cohort level this equals 18/169 (10.7%) of those imaged and 18/216 (8.3%) of all enrolled. Notably, 6/30 (20.0%) of infants requiring a follow-up echocardiogram did not return, which corresponds to 6/169 (3.6%) of the analyzed cohort.

• It is not appropriate to compare the study’s findings with prevalence estimates derived from a passive surveillance system (line 284). Passive surveillance generally underestimates prevalence and identifies mostly severe cases, while active case ascertainment (as in this study) detects a broader spectrum, including minor anomalies.

We addressed this important comment more carefully in our Discussion:

“A smaller case series identified CHDs in 2/18 infants, with one having complex disease consisting of pulmonary venous return, total atrioventricular septal defect, and a persistent PDA (32). For background, CHDs have been reported to occur in approximately 5 to 7 per 1000 live births in Brazil (28), with a higher prevalence observed in infants with low birth weight (34). Of note, these figures are derived from passive surveillance systems that preferentially capture more severe cases and under-ascertain milder lesions, particularly in regions with limited diagnostic capacity. Generally, accurate tracking of cardiac defects in Brazil may be difficult given the notable rates of underreporting, especially in the northern and northeastern regions of the country (28). While the advent of the Unified Health System has increased health care access for Brazilians, there are still challenges in achieving a similar universal healthcare model across the country (35). We suspect that the regions with the highest rates of underreporting are reflective of the discrepancy in healthcare infrastructure and access to pediatric cardiology services, and thus comparisons to population-based analyses must take this into consideration.”

• The association between ZIKV exposure and cardiac anomalies appears overstated. Hoffman and Kaplan (2002) showed that while severe congenital heart disease occurs in ~1% of cases, inclusion of minor anomalies raises prevalence to ~7.5%. This context should be acknowledged.

We cited Hoffman and Kaplan (2002) and included the context that the prevalence of CHD may be higher if minor anomalies are included.

• The recommendation for routine fetal echocardiograms in ZIKV-exposed pregnancies may not be justified. Such practice could add unnecessary stress for families, increase healthcare costs, and may not be clinically warranted, especially given that most anomalies (approximately 75%, as stated) resolved spontaneously. Microcephalic infants warrant closer follow-up, but routine echocardiography for all does not appear supported by the data, particularly since only 7.7% (4/52) of microcephalic patients had persistent anomalies after two echocardiograms, and their clinical significance (symptoms, treatment) is not described.

We have modified our recommendations to the following:

“In hospital settings with robust cardiovascular infrastructure, echocardiogram should be considered in all children with CZS as a screening method to identify structural cardiac lesions. This approach is analogous to screening protocols utilized in other congenital infections, such as long bone radiographic evaluations in syphilis (49) and hearing assessments in cytomegalovirus (CMV) infection (50). In regions with constrained resources, a more nuanced and context-specific strategy may be warranted. Patients who are normocephalic at birth may follow the same criteria as those established for the general population of mothers and infants, which includes a complete physical exam and measure of oxygen saturation. However, patients with microcephaly should be considered for a post-natal echocardiogram, if resources are permitting. We believe the importance of cardiac screening is underscored by the markedly higher mortality observed in children with CZS compared with those without the syndrome, where the leading causes of death are cardiac in nature (51).

• The limitations section should be expanded, particularly to address the modest sample size, which may explain why only microcephaly was associated with cardiac anomalies.

Weh have expanded our limitations to the following:

“However, there are several limitations to acknowledge. First, we lacked a non-exposed control group, as this was a clinic-based cohort assembled during a public health emergency. Notably, recruiting controls and screening asymptomatic women was not technically feasible during the epidemic given that healthcare resources were heavily strained by the large number of symptomatic referrals. Therefore, our estimates of about 18% abnormal echocardiograms overall, and a higher abnormal rate among MC than NC infants, are benchmarked to population norms rather than infants imaged under identical protocols. Second, selection/referral bias is possible because of 216 exposed infants with postnatal follow-up, 169 (78%) underwent echocardiography, and recruitment at a tertiary pediatric center may result in more complex phenotypes. Third, we acknowledge that follow-up was incomplete, which might have resulted in an underpowered analysis of persistent CV abnormalities: of the 30 infants with initial abnormalities, 24 returned and roughly one quarter had persistent lesions. Additionally, distinguishing physiologic from pathologic findings is challenging given age heterogeneity at imaging (initial 86 ± 80 days; repeat 188 ± 169 days). Finally, we recognize that none of the defects were found to have major clinical repercussions in early childhood, thus the impact of this increased rate of cardiac lesions still needs to be explored.

We designed our follow up study with these limitations in mind. Despite the absence of a non-exposed control group, we included an internal MC-NC comparison under identical protocols, complemented by multivariable analyses adjusting for gestation, sex, birth weight, delivery mode, trimester of infection, maternal age, hypertension, and diabetes, which still supported an association between MC and cardiac anomalies. Echocardiography was proactively offered to all patients with laboratory-confirmed ZIKV exposures, thus reducing selective imaging by symptom severity. Moreover, imaging acquisition and interpretation were performed by pediatric cardiologists using standardized protocols. Lastly, to minimize the challenges of differentiating between physiologic and pathologic findings, we standardized and prespecified physiologic windows (e.g., PDA in term infants ≤ 15 days and in preterm infants ≤ 3 months; PBS normal in preterm) and performed sensitivity analyses incorporating age at echocardiography. Although these measures do not completely eliminate the limitations described above, they improve the study’s internal validity and highlight the increased prevalence of structural cardiac abnormalities in ZIKV-exposed infants, especially among those with MC.”

Reviewer #3:

The conclusions overstate the implications of the findings. The statement that CHD 'should be considered a determinant of CZS' is premature. The evidence is limited to a modest statistical association (OR = 3.40; 95% CI: 1.15–10.02), which is imprecise and based on small numbers. It would be more accurate to frame this as a hypothesis: that severe neurological involvement and cardiac abnormalities may reflect a shared vulnerability of developing organ systems to viral or inflammatory insults during organogenesis. The transient nature of most lesions should also be acknowledged while their initial presence may indicate systemic developmental disturbance, the majority resolve and appear clinically mild.

We have modified our statement to the following:

“We hypothesize that these cardiac and neurologic anomalies may reflect a shared vulnerability secondary to viral or inflammatory insults during organogenesis. The presence of cardiac lesions may suggest a systemic developmental disturbance; however it is important to acknowledge that the majority resolve and appear to be clinically mild. Regardless, given the increased rates of cardiac findings associated with vertical transmission of ZIKV, we build upon our prior findings, and propose a consideration of the diagnostic criteria of CZS to include CHDs.”

**Editorial and Data Presentation Modifications?**

Use this section for editorial suggestions as well as relatively minor modifications of existing data that would enhance clarity. If the only modifications needed are minor and/or editorial, you may wish to recommend “Minor Revision” or “Accept”.

Reviewer #1:

Line 55 should likely be most of the CV defects resolved over time and at follow up.
Line 62: Suggest: We conducted a follow-up to evaluate cardiologic findings of infants from such mothers.
Line 72: delete (78%) as irrelevant to study
Line 73: Thirty (17.8%) had cardiac anomalies [consisting of ...] that were detected by imaging (?)
Line 77: presence of [persistent?] CV defects
Line 82 and elsewhere: Using the term “most defects” seems misleading since that was not the case in 25% of patients – in a majority of patients might be better
Line 112: “our group” is not a citation – cite first author et al.
Table 1: Instead of having a footnote for abbreviations, incorporate into table itself. Same for Table 3.

Thank you for your comments. All suggested modifications have been addressed except for the “presence of [persistent?] CV defects” since our linear regression model evaluated initial echocardiograms, not follow up echocardiograms

Reviewer #2:

• Line 68: Define the abbreviation “CV” in the abstract.
• Line 103: RT-PCR abbreviation was already defined previously and need not be repeated.
• Lines 99–101: The description of ZIKV symptoms in adults is not directly relevant to the present study and could be omitted.
• Line 343: Typographical error – “chilhoood” should read “childhood.”
• Figure 2 is misleading when using relative percentages, as it appears that more anomalies were detected during follow-up when in fact the opposite occurred. A revised figure should use absolute numbers and clearly state that 75% of anomalies detected initially resolved on follow-up.
• Line 257: The sentence should be rephrased as: “The multivariate logistic regression analysis suggests that microcephaly is associated with cardiac anomalies” rather than “microcephaly as a predictor of cardiac anomalies.”

Thank you for your comments. All suggested modifications have been addressed and figure 2 was revised to reflect absolute numbers

Reviewer #3:

• Add a clearly defined research question and explicit study objectives in the Introduction.

We clarified the research question and objectives in the Introduction section:

“In this longitudinal assessment, our research questions were: what is the prevalence and spectrum of structural cardiac abnormalities; do these risks differ by cephalic status at birth; and what proportion of anomalies persist on repeat echocardiography? Accordingly, our objectives were to (i) estimate the prevalence of any structural cardiac abnormalities in early infancy; (ii) compare prevalence and abnormality patterns between MC and normocephalic (NC) infants; (iii) characterize lesion-specific frequencies; (iv) determine the proportion of defects that resolve versus persist on follow-up; and (v) explore clinical and perinatal predictors of cardiac abnormalities.”

• Revise Table 1 and Table 2 to include p-values, denominators, and clearer outcome structure.

Table 1 was revised to include p values

Table 2 was revised to include p values and denominators

• Improve figure legends and include 95% confidence intervals in graphical results.

Figure 2 was revised to reflect absolute numbers. Given the extremely small size in many of the categories in the figure (e.g. NC abnormal minor repeat n = 1), adding 95% confidence intervals would clutter the figure and reduce its clarity.

• Correct typographical 'childhoood' l.343).

The typographical error was corrected

• Provide a detailed section on study limitations in the Discussion, including: lack of non-exposed controls, selection bias, small sample sizes for persistent lesions, and challenges in interpreting physiologic vs pathologic findings by age and discuss how theses limitations may affect the conclusions if the study

We have expanded our limitations section to address the concerns as mentioned above.

“However, there are several limitations to acknowledge. First, we lacked a non-exposed control group, as this was a clinic-based cohort assembled during a public health emergency. Notably, recruiting controls and screening asymptomatic women was not technically feasible during the epidemic given that healthcare resources were heavily strained by the large number of symptomatic referrals. Therefore, our estimates of about 18% abnormal echocardiograms overall, and a higher abnormal rate among MC than NC infants, are benchmarked to population norms rather than infants imaged under identical protocols. Second, selection/referral bias is possible because of 216 exposed infants with postnatal follow-up, 169 (78%) underwent echocardiography, and recruitment at a tertiary pediatric center may result in more complex phenotypes. Third, we acknowledge that follow-up was incomplete, which might have resulted in an underpowered analysis of persistent CV abnormalities: of the 30 infants with initial abnormalities, 24 returned and roughly one quarter had persistent lesions. Additionally, distinguishing physiologic from pathologic findings is challenging given age heterogeneity at imaging (initial 86 ± 80 days; repeat 188 ± 169 days). Finally, we recognize that none of the defects were found to have major clinical repercussions in early childhood, thus the impact of this increased rate of cardiac lesions still needs to be explored.

We designed our follow up study with these limitations in mind. Despite the absence of a non-exposed control group, we included an internal MC-NC comparison under identical protocols, complemented by multivariable analyses adjusting for gestation, sex, birth weight, delivery mode, trimester of infection, maternal age, hypertension, and diabetes, which still supported an association between MC and cardiac anomalies. Echocardiography was proactively offered to all patients with laboratory-confirmed ZIKV exposures, thus reducing selective imaging by symptom severity. Moreover, imaging acquisition and interpretation were performed by pediatric cardiologists using standardized protocols. Lastly, to minimize the challenges of differentiating between physiologic and pathologic findings, we standardized and prespecified physiologic windows (e.g., PDA in term infants ≤ 15 days and in preterm infants ≤ 3 months; PBS normal in preterm) and performed sensitivity analyses incorporating age at echocardiography. Although these measures do not completely eliminate the limitations described above, they improve the study’s internal validity and highlight the increased prevalence of structural cardiac abnormalities in ZIKV-exposed infants, especially among those with MC.”

**Summary and General Comments**


Use this section to provide overall comments, discuss strengths/weaknesses of the study, novelty, significance, general execution and scholarship. You may also include additional comments for the author, including concerns about dual publication, research ethics, or publication ethics. If requesting major revision, please articulate the new experiments that are needed.

Reviewer #1: The study by Dang and colleagues is important and needs to be published, but the manuscript needs considerable revision before being acceptable for PLOS NTD. Below are a number of suggestions that would enhance clarity and readability. The single item that very much needs attention relates to the timing of followup and the conclusion that MC infants have a higher rate of persistence of CV abnormalities than NC infants. The problem is that, unless I somehow missed it, there is no analysis of this conclusion relative to the age of followup. The followup was conducted within 1-12 months (184 ± 139 days), which is a huge interval. Was that interval somewhat similar in MC and NC infants? In other words, the way it is currently written it is unclear whether their might be significant bias in the age at followup between MC and NC infants.

Reviewer #2: This study adds valuable information regarding ZIKV and cardiac anomalies. However, the manuscript requires revision to avoid overstating findings, to provide additional methodological detail, and to strengthen the discussion of clinical relevance and limitations.

Reviewer #3: This manuscript addresses an important and under-researched aspect of congenital Zika syndrome. The longitudinal design and careful definition of exposure are strong features, and the findings are potentially relevant for clinical monitoring and hypothesis generation. However, the study is limited by methodological weaknesses, particularly the absence of a control group, potential selection bias, and small numbers driving the main associations. The interpretation currently overreaches the strength of the evidence. I recommend major revisions to clarify the objectives, strengthen data presentation, and temper conclusions. The addition of a well-documented 'Limitations' section in the Discussion is essential.
